# *Staphylococcus aureus* and MRSA Growth and Biofilm Formation after Treatment with Antibiotics and SeNPs

**DOI:** 10.3390/ijms161024656

**Published:** 2015-10-16

**Authors:** Kristyna Cihalova, Dagmar Chudobova, Petr Michalek, Amitava Moulick, Roman Guran, Pavel Kopel, Vojtech Adam, Rene Kizek

**Affiliations:** 1 Department of Chemistry and Biochemistry, Mendel University in Brno, Zemedelska 1, CZ-613 00 Brno, Czech Republic; E-Mails: kriki.cihalova@seznam.cz (K.C.); dagmar.chudobova@centrum.cz (D.C.); petrmichalek85@gmail.com (P.M.); amitavamoulick@gmail.com (A.M.); r.guran@email.cz (R.G.); paulko@centrum.cz (P.K.); vojtech.adam@mendelu.cz (V.A.); 2 Central European Institute of Technology, Brno University of Technology, Technicka 3058/10, CZ-616 00 Brno, Czech Republic; 3 Department of Microelectronics, Faculty of Electrical Engineering and Communication, Brno University of Technology, Technicka 3058/10, CZ-616 00 Brno, Czech Republic

**Keywords:** *Staphylococcus aureus*, methicillin-resistant *Staphylococcus aureus*, antibiotics, selenium nanoparticles

## Abstract

Methicillin-resistant *Staphylococcus aureus* (MRSA) is a dangerous pathogen resistant to β-lactam antibiotics. Due to its resistance, it is difficult to manage the infections caused by this strain. We examined this issue in terms of observation of the growth properties and ability to form biofilms in sensitive *S. aureus* and MRSA after the application of antibiotics (ATBs)—ampicillin, oxacillin and penicillin—and complexes of selenium nanoparticles (SeNPs) with these ATBs. The results suggest the strong inhibition effect of SeNPs in complexes with conventional ATBs. Using the impedance method, a higher disruption of biofilms was observed after the application of ATB complexes with SeNPs compared to the group exposed to ATBs without SeNPs. The biofilm formation was intensely inhibited (up to 99% ± 7% for *S. aureus* and up to 94% ± 4% for MRSA) after application of SeNPs in comparison with bacteria without antibacterial compounds whereas ATBs without SeNPs inhibited *S. aureus* up to 79% ± 5% and MRSA up to 16% ± 2% only. The obtained results provide a basis for the use of SeNPs as a tool for the treatment of bacterial infections, which can be complicated because of increasing resistance of bacteria to conventional ATB drugs.

## 1. Introduction

The formation of biofilms is a natural property of a wide range of bacterial species [1,2]. These species can cause many serious bacterial infections initiating serious complications [3,4,5]. Staphylococci are recognized as the most frequent causes of biofilm-associated infections [6], dental plaque [7] and this exceptional status among biofilm-associated pathogens is due to the fact that they are frequent commensal bacteria on the human skin and mucous surfaces (and those of many other mammals).

Excessive use of methicillin antibiotics (ATBs) led to the formation of methicillin-resistant *S. aureus* (MRSA) with adhesion properties. The occurrence of resistant strains of bacteria is a complication of all medical practices that are commonly encountered in recent time with CA-MRSA (community-associated methicillin-resistant *S. aureus*) [8] and HA-MRSA (hospital-acquired methicillin-resistant *S. aureus*) [9]. These strains are unlike non-resistant *S. aureus* resistant to β-lactam ATBs [10]. All MRSA strains carry an acquired genetic determinant-*mecA* or *mecC*- which encodes low affinity penicillin binding proteins-PBP2a [11]. The *mecA* gene is present on a Staphylococcal cassette chromosome *mec* (SCC*mec*), which is a genomic island that concentrates β-lactam ATBs resistance genes and other resistance genes [12]. The majority of MRSA found in clinical testing are multidrug resistant (MDR) [13]. MRSA may be resistant to other groups of antibiotics such as aminoglycosids, cefalosporins, penicillins or glycopeptides. Included in the glycopeptides group is vancomycin, which has long been considered the antibiotic of last resort against serious and multi-drug-resistant infections caused by Gram-positive bacteria. However, vancomycin resistance has emerged, first in enterococci [14,15] and, more recently, in *Staphylococcus aureus* [16].

Because of an increasing resistance of bacterial species to ATBs, it is necessary to develop new methods for bacterial inhibition. Recently, scientists were increasingly focused on the activity of silver nanoparticles that exhibit antibacterial, antiviral and antifungal effects [17], as in the case of gold nanoparticles [18], TiO_2_ nanoparticles [19], or a mixture of Ag/ZnO nanoparticles [20]. A study [21] compared the antibacterial effect between silver and selenium nanoparticles; antibacterial effects of selenium nanoparticles inhibited the bacteria *S. aureus* surprisingly better than nanoparticles of silver phosphate. In another study, selenium nanoparticles (SeNPs) showed good properties as antibacterial drugs. The effect of selenium nanoparticles was confirmed in a study by Tran *et al.* [22] that showed that the growth of *S. aureus* is inhibited after three hours of incubation with SeNPs. The combination of ATBs drugs, which are aimed at groups of multi-drug resistant bacteria [23], with the metal nanoparticles can also represent a new alternative as pharmaceutical tools with a high antibacterial effect on a broad spectrum of both resistant and non-resistant bacteria [24]. Metal nanoparticles interacting with cellular components (DNA, RNA and ribosomes) deactivate and effectively alter cellular processes [25]. Metal nanoparticles penetrate the cell membrane to reach the cytosol due to their ability to dissolve slowly while releasing ions, but the exact mechanism of the metal nanoparticles antimicrobial action remains unclear [21].

For determining antibacterial effect to resistant bacteria we compared non-resistant *S. aureus* and methicillin-resistant *S. aureus*. This study examined the changes on the cellular level in cultures of *S. aureus* and MRSA after incubation with ATBs and complexes of SeNPs with ATBs. At the same time, attention was focused on the changes of biofilm formation after ATBs and complexes of SeNPs with ATBs treatment. Real-time cell analysis (RTCA) on xCELLigence device was used for this determination [26,27]. The method works on the principle of cell adhesion on the surface of electrodes, which modulates the resulting impedance [28]. In the case of bacteria adherence of biofilm on the surface of the electrodes occurs [29] and thereby the change of the relative impedance is observed [30]. The study was supported through monitoring of the activity of the expression process of ATB resistance genes.

## 2. Results and Discussion

### 2.1. Influence of Antibacterial Compounds to Growth Properties

**Figure 1:**
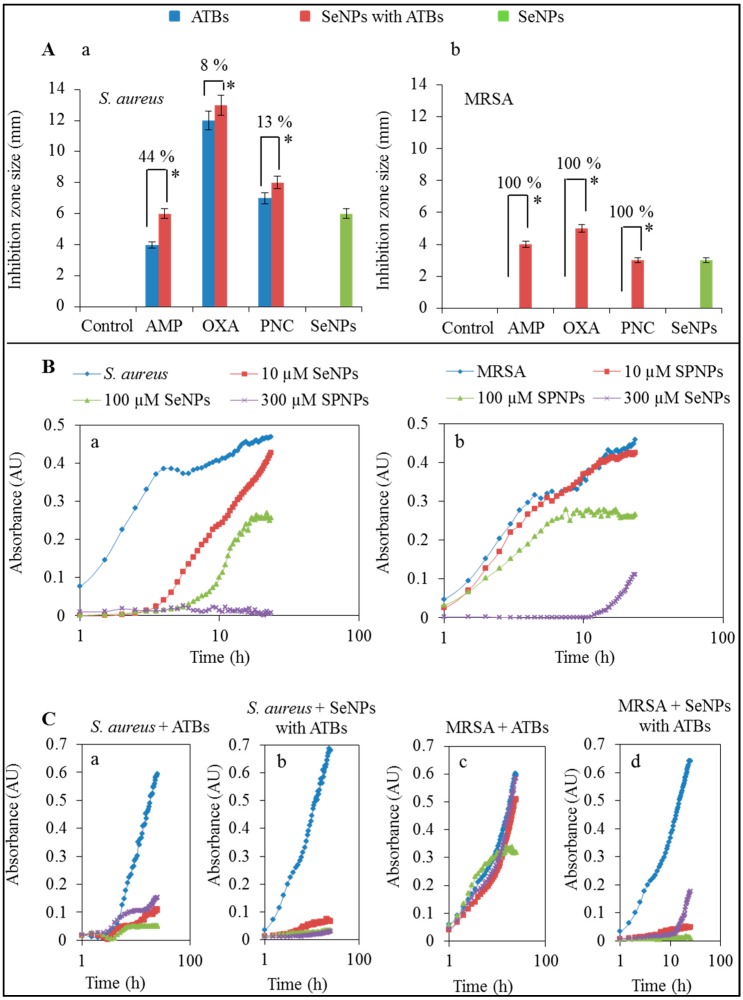
(**A**) Determination of inhibition zones after application of circular discs on the *S. aureus* (**a**) and MRSA (**b**) strains with 100 µM concentration of ampicillin, oxacillin, penicillin or complexes (100 µM of nanoparticles and 100 µM of ATBs) of SeNPs with ampicillin, SeNPs with oxacillin and SeNPs with penicillin. Cultivation was carried out at 37 °C for 24 h; (**B**) Optimization of SeNPs concentration for non-resistant *S. aureus* (**a**) and MRSA (**b**) for other measurements; (**C**) Growth curves after application of ATBs (ampicillin—red line, oxacillin-green line, penicillin—purple line) on *S. aureus* (**a**) and MRSA (**c**)—blue line and complexes of SeNPs (100 μM) with the same ATBs (100 μM) *S. aureus* (**b**) and MRSA (**d**)—blue line. All data represent mean ± S.D. NS, not significant, * *p* < 0.05.

Microbiological determination of the inhibition zone sizes showed evident inhibitory effect resulting from the application of SeNPs enhanced by forming a complex with ampicillin, oxacillin and penicillin. The ATBs alone demonstrated antibacterial properties only for sensitive *S. aureus* with sizes of the growth inhibition zones within the range of 4–12 mm (Figure 1A). However, the complexes of SeNPs with ATBs showed the significant antibacterial effect with inhibition zone sizes within the range of 6–13 mm for sensitive *S. aureus* (Figure 1(Aa)) and 3–5 mm for MRSA (Figure 1(Ab)). After the application of ampicillin, the observed sizes of growth inhibition zones were 4 and 6 mm for non-resistant *S. aureus* (Figure 1(Aa)) and 0 and 4 mm for MRSA (Figure 1(Ab)). Application of oxacillin provided the highest growth inhibition zones with sizes of 12 and 13 mm for non-resistant *S. aureus* (Figure 1(Aa)) and 0 and 5 mm for MRSA (Figure 1(Ab)). In the case of penicillin, the sizes of inhibition zones were 7 and 8 mm for non-resistant *S. aureus* (Figure 1(Aa)) and 0 and 3 mm for MRSA (Figure 1(Ab)). Application of other drugs exhibited similar results. Although MRSA did not form inhibition zones after application of the discs containing ATBs without SeNPs, complexes of SeNPs with ATBs manifested inhibition zones between 4–6 mm. Application of complex of SeNPs with AMP, OXA, PNC has about 0%, 25%, 54% higher inhibition effect than SeNPs alone for non-resistant *S. aureus*, respectively, and about 25%, 40%, 0% higher inhibition effect than SeNPs alone for MRSA, respectively. The inhibition effect of SeNPs is about 50% higher for non-resistant *S. aureus* than for MRSA. In the case of non-resistant *S. aureus*, larger inhibition zones were observed after the application of complexes of SeNPs with ATBs than ATBs alone. In the non-resistant *S. aureus* case, ampicillin, oxacillin and penicillin caused higher inhibitory effects (44%, 8% and 13% respectively) when applied in combination with SeNPs than ATBs alone (Figure 1(Aa)). Application of ATBs in combination with SeNPs caused 100% higher inhibition effect because ATBs do not cause formation of the inhibition zones (Figure 1(Ab)). Muhsin *et al.* [31] reported that the application of silver nanoparticles caused a 17 mm wide growth inhibition zone for *S. aureus*. On the other hand, the use of gentamycin alone could cause a growth inhibition zone of larger size (31 mm). However, the modification of silver nanoparticles with gentamycin showed a small increase of the inhibition zone width up to 33 mm [31].

The antibacterial activity of ATBs or their complexes with SeNPs after 24 h was confirmed by the method of the growth curves [21]. The 50% inhibitory concentration was determined in the previous study [21] and for our study, 100 µM concentrations of SeNPs in combination with 100 µM of ATB (Figure 1(Ba,b)) was used. Concentration of SeNPs that showed inhibitory differences between non-resistant *S. aureus* and MRSA were selected for measurements in this paper. ATBs concentrations were chosen on the basis of previous measurements. For the comparison of ATBs with or without SeNPs, we used only one concentration (100 µM) of ATBs, and it was found that the ATBs applied in combination with SeNPs (100 µM) showed a greater antibacterial effect than ATBs alone. The inhibition effect of ATBs was significant on a non-resistant *S. aureus* (Figure 1(Ca)). But in the case of MRSA, ATBs, as expected, were mostly ineffective (Figure 1(Cc)). Only oxacillin showed low antibacterial activity with MRSA without antibacterial compounds. The application of complexes of SeNPs with ATBs caused almost complete inhibition of both strains (non-resistant *S. aureus* and MRSA) in the case of all 3 types of applied ATBs-ampicillin, oxacillin, penicillin (Figure 1(Cb,d)).

### 2.2. Influence of Antibacterial Compounds to Biofilm Formation

The assessment of the antimicrobial components was further carried out to test the viability of the cells. The relative impedance depending on the adherence of cell culture to the gold electrodes in real time was used for this purpose. Real time xCELLigence analysis system is an impedance-based cell detection platform that provides a non-invasive, label-free way for continuous cellular monitoring [32,33,34]. This method has been used in many published research studies [27,35,36,37]. Junka *et al.* [28] showed that xCELLigence system can also be useful for microbiological tests, including (i) measurements of morphological changes in prokaryotic cells; (ii) measurement of bacterial biofilm formation and (iii) impact of antiseptics on the biofilm structure.

**Figure 2:**
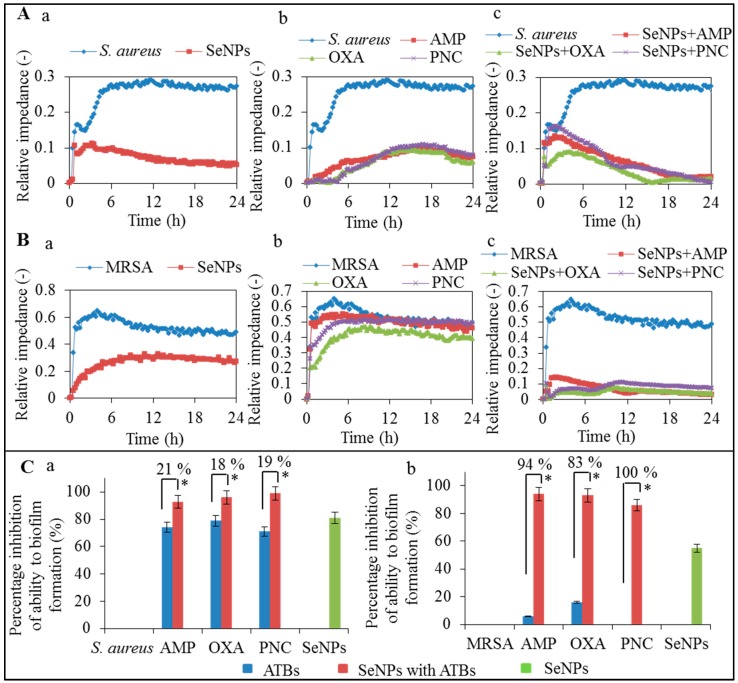
(**A**) Monitoring of biofilm disruption after application of 100 μM SeNPs on *S. aureus*—blue line (**a**) and ATBs (100 μM): ampicillin—red line, oxacillin—green line, penicillin—purple line on *S. aureus*—blue line (**b**) and complexes of SeNPs (100 μM) with the same ATBs (100 μM) on *S. aureus*—blue line (**c**); (**B**) Monitoring of biofilm disruption after application of 100 μM SeNPs on MRSA—blue line (**a**) and ATBs (100 μM) ampicillin—red line, oxacillin—green line, penicillin—purple line on MRSA—blue line (**b**) and complexes of SeNPs (100 μM) with the same ATBs (100 μM) on MRSA—blue line (**c**); (**C**) Comparison of differences in relative impedance after application of 100 μM concentration of ATBs or complexes of ATBs with SeNPs (100 μM) and SeNPs alone (100 μM) on *S. aureus* (**a**) and MRSA (**b**) after 24 h of measurement. All data represent mean ± S.D. from three measurements, NS, not significant, * *p* < 0.05.

Figure 2A depicts the application of SeNPs on the non-resistant *S. aureus* and MRSA, showing that the biofilm formation was more hampered in presence of SeNPs than in control (*S. aureus* or MRSA without application of antibacterial components). The xCELLigence suitability for the microbiological tests was confirmed in the same way as in the study previously conducted by Junka *et al.* [28]. The tested bacteria must always be able to adhere on the surface of electrode at the bottom in the measuring well [7,38]. The ability to destroy the biofilm formation is one of the virulence factors in bacteria with low sensitivity to ATBs [39].

After application of the antibacterial agents, the biofilm is disrupted and bacterial cells are released from the surface of electrodes. This trend is measured as an impedance values and depicted in the graph (Figure 2A,B). The differences in the relative impedance of *S. aureus* showed decreases in the values of all components applied in comparison with the control, which is caused by balanced violation of the biofilm formed on the electrode surface. In the case of non-resistant *S. aureus*, ATBs and complexes of SeNPs with ATBs disrupted the bacterial biofilm, and after that a higher effect on these bacteria could be seen (Figure 2A). The biofilm formed by MRSA (Figure 2(Bb,c)) was more resistant to ATBs and complexes of SeNPs with ATBs than the biofilm formed by the non-resistant *S. aureus* (Figure 2(Ab,c)). Figure 2B shows the percentage decrease of biofilm formation after application of ATBs and complexes of SeNPs with ATBs in comparison with control. The control sample of non-resistant *S. aureus* reached the relative impedance values of 0.29 and the control sample of MRSA reached 0.52 after 24-h measurement and from these values the decrease of the relative impedance after application of ATBs and complexes of SeNPs with ATBs was calculated. The measurements were performed in triplicates. Low adhesion showed a decreasing trend caused by the biofilm formation on the electrodes, and from this we can conclude that the application of ATBs and complexes of SeNPs with ATBs caused disruption of the biofilm on the electrodes. The results are summarized in Table 1.

**Table 1:** Percentage disruption of biofilm after treatment of antibacterial component (ATBs, SeNPs + ATBs) after 24 h (Figure 2(Ca,b)).

Compounds	Biofilm Disruption (%)			
	*S. aureus*		MRSA	
	ATB	SeNPs + ATBs	ATB	SeNPs + ATBs
AMP	74 ± 2	93 ± 3	6 ± 5	94 ± 4
OXA	79 ± 5	96 ± 2	16 ± 2	93 ± 4
PNC	71 ± 2	99 ± 7	0	86 ± 2
SeNPs	81 ± 4		55 ± 3	

The application of complex of SeNPs with ATBs (SeNPs with ampicillin, SeNPs with oxacillin, SeNPs with penicillin) caused a twofold decrease in the relative impedance compared to ATBs only. For non-resistant *S. aureus* the significantly higher effect of SeNPs with ATBs was not observed, however increased inhibition of biofilm formation in case of complexes of SeNPs with ATBs was confirmed.

### 2.3. Determination of Expression Intensity of mecA Gene

The *mecA* gene is responsible for bacterial resistance to β-lactam ATBs and occurs at staphylococcal chromosome cassette *mec* (SCC*mec*), which besides *mecA* gene contains a number of other genes causing the resistance [10]. Its expression was monitored after application and also after 24-h cultivation in the presence of ATBs alone (50 µM) or at same concentration of ATBs in complexes with SeNPs (100 µM). Bacterial strain MRSA even without drugs application always exhibited the expression of this gene in comparison with the strain of *S. aureus*, where this expressed gene was absent.

**Figure 3:**
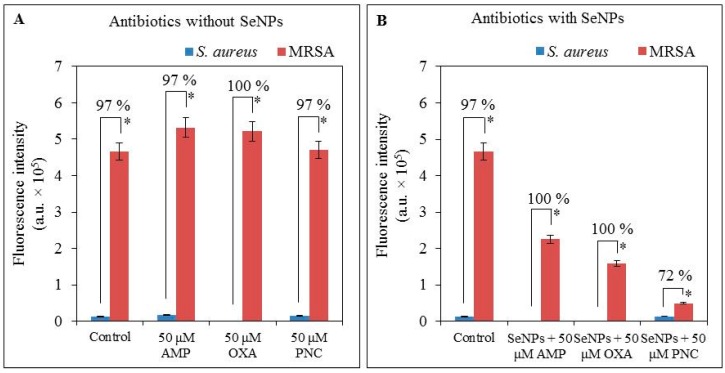
Monitoring of *mecA* gene expression in bacterial strains *S. aureus* and MRSA after 24 h of growth with (**A**) 50 µM ATBs concentration and with (**B**) 100 µM concentration of SeNPs with 50 µM ATBs concentration in complexes using PCR and subsequent gel electrophoresis. Controls are bacterial strains (*S. aureus* and MRSA) without application of antibacterial component. All data represent mean ± S.D. NS, not significant, * *p* < 0.05.

Expression of the *mecA* gene in MRSA without antibacterial compounds was higher (by 84%) than the standard expression of the *16S* gene. Fluorescence values of *16S* (the housekeeping gene with a luminescence level 13,098 a.u. (absorbance units) for non-resistant *S. aureus* and 12,544 a.u. for MRSA) ware subtracted from the *mecA* (Figure 3A).

For non-resistant *S. aureus* the expression of *mecA* gene reached the intensity of fluorescence of 13,059 a.u. while for MRSA was the fluorescence intensity much higher (36 times). *S. aureus* grew only in the presence of 50 µM ampicillin and penicillin, but not in the presence of 50 µM concentration of oxacillin. For ampicillin and penicillin, non-resistant *S. aureus* reached a very low expression of *mecA* gene (17,315 and 15,543 a.u., respectively). MRSA showed higher expression by 14% in the case of using 50 µM concentrations of ampicillin or oxacillin, and low expression of *mecA* gene after 24 h cultivation with 50 µM penicillin, when comparing with MRSA without application of antibacterial compounds (Figure 3A).

In MRSA, the expression of the *mecA* gene was decreased after application of ATBs with selenium nanoparticles when compared with expression of the *mecA* gene in MRSA without antibacterial compounds. Higher fluorescence intensity of *mecA* gene expression was observed in MRSA after incubation with 50 μM of ATBs (ampicillin, oxacillin and penicillin) alone (Figure 3), as compared to complexes of SeNPs with ATBs, for which the fluorescence intensities of expression were 48%, 70% and 90% lower, respectively (Figure 3B).

In non-resistant *S. aureus*, *mecA* gene expression was observed to be 37% higher after incubation with 50 µM concentration of ampicillin in comparison with control (*S. aureus* without antibacterial compounds). Non-resistant *S. aureus* did not grow after application of oxacillin (50 µM) thus, gene expression could not be determined (Figure 3B). In the case of ATBs with selenium nanoparticles, the growth of non-resistant *S. aureus* was observed only after application of 50 µM concentration of penicillin (Figure 3B). The expression was measurable only in the case of penicillin. This expression was by 7% higher compared to non-resistant *S. aureus* without antibacterial compounds.

*MecA* gene expression in resistant strains of bacteria was discussed in the study of Rudkin *et al.* [40], where the use of different concentrations of oxacillin increased the level of expression of *mecA* gene and they detected a higher level of toxicity in CA-MRSA than in HA-MRSA. It was observed, that in the HA-MRSA, high expression of PBP2a reduced the toxicity by disrupting the *agr* quorum sensing system, which controls the expression of virulence.

### 2.4. Determination of Changes in Protein Structure

**Figure 4:**
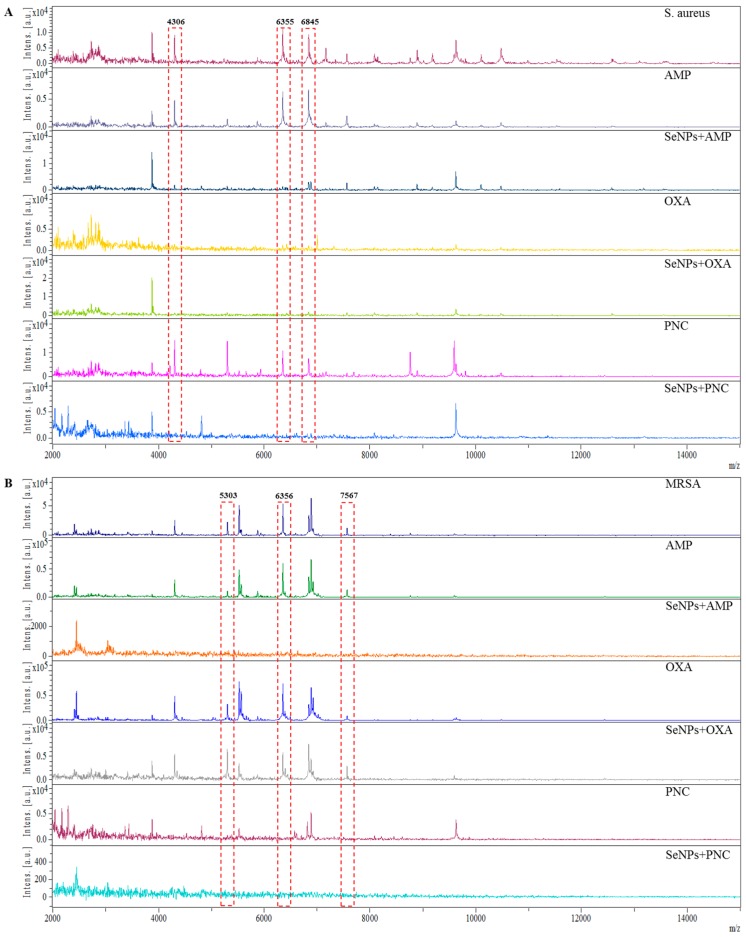
The comparison of MALDI-TOF mass spectra of (**A**) *S. aureus* and (**B**) MRSA with ampicillin, SeNPs with ampicillin, oxacillin, SeNPs with oxacillin, penicillin and SeNPs with penicillin. The analysis was performed in linear positive mode. A solution of 2,5-dihydroxybenzoic acid (concentration 20 mg·mL^−1^) in 30% acetonitrile and 0%, 1% trifluoroacetic acid was used as a matrix. The laser power was set to 75%. The highlighted peaks show the biggest differences in compared spectra.

The significant changes in the protein composition of bacterial strains caused by the effect of selenium nanoparticles were observed using mass spectrometry. In the mass spectra of non-resistant *S. aureus* with different ATBs, three peaks with *m*/*z* 4306, 6355 and 6845 were selected as significant peaks showing the differences between mass spectra (Figure 4A). Similarly, in the mass spectra of MRSA, peaks with *m*/*z* 5303, 6356 and 7567 were selected (Figure 4B). Peaks with the same or similar *m*/*z* value were described in various publications [41,42]. It is obvious that the presence of selenium nanoparticles causes distinct changes in protein profiles—according to the mass spectra, the most efficient were SeNPs with oxacillin in the case of non-resistant *S. aureus* (Figure 4A) and with ampicillin and penicillin in the case of MRSA (Figure 4B). These compounds caused the suppressed expression of almost all proteins compared to the control strain of non-resistant *S. aureus* and MRSA without the addition of SeNPs (Figure 4A,B). These results can be a suitable basis for comparison of the protein representation and their effect on the pathogenicity of bacteria.

These protein changes may affect the pathogenicity of bacteria. Interaction of metal ions with DNA can affect the protein-DNA interactions [43]. In the case of silver nanoparticles, the interaction with DNA or proteins can occur through Ag-N bonding [44] and selenium can assume a similar mechanism of bonding to DNA. This process in prokaryotes regulates the expression of a number of genes in virulence and pathogenesis [45]. Similarly, the study of Gopal *et al.* [46] refers to the changes in the protein profile of bacteria measured by mass spectrometry in bacterial cultures of *S. aureus* and *Pseudomonas aeruginosa*, which were incubated for 24 h with Ag, ZnO, TiO_2_, NiO and Pt nanoparticles. Between 2–6 h of incubation, no change in the signal was observed, while rapid decrease of signal occurred between 12–24 h of incubation with Ag, ZnO and TiO_2_ nanoparticles in both tested bacterial strains in comparison with the control. After the application of NiO NPs and Pt NPs, no changes were observed.

This test determined minimum inhibitory concentration of individual substance for non-resistant *S. aureus* and MRSA and from these values were calculated FICI (fractional inhibitory concentration index) indicating the synergy or antagonism of two substances. For *S. aureus*, the values of FICI were for SeNPs with AMP and SeNPs with PNC was partly synergistic and for SeNPs with oxacillin had additive effect. In the case of MRSA, complexes of SeNPs with AMP and SeNPs with PNC were determined the FICI as 0.53 and for SeNPs with OXA 0.57 indicating partial synergy between the SeNPs and ATBs (Table 2). These results are very important for inhibition of resistant bacteria, because a synergism occurs between the SeNPs and ATBs, due to that bacteria in the sample are inhibited by the effect of both substances in the complex. The results thus suggest that the combinations of SeNPs with ATBs exhibited improved inhibition of methicillin-resistant bacteria with partial synergy or additive effect. Drugs were assayed separately, thus confirming the hypothesis that antibiotic-resistant inhibitors combined with antibiotics are a potential method for solving the problem caused by resistant bacteria.

**Table 2:** Minimum inhibitory concentration and FICI values of SeNPs with antibiotics.

Strain	MIC (µM)				FICI		
	AMP	OXA	PNC	SeNPs	SeNPs + AMP	SeNPs + OXA	SeNPs + PNC
*S. aureus*	50	25	50	10	0.70	0.90	0.70
MRSA	300	150	300	20	0.53	0.57	0.53

## 3. Experimental Section

### 3.1. Cultivation of S. aureus and MRSA

*S. aureus* (NCTC 8511) and MRSA (ST239) were obtained from the Czech Collection of Microorganisms, Faculty of Science, Masaryk University, Brno, Czech Republic. Cultivation media (LB = Luria Bertani) were inoculated with bacterial culture and were cultivated for 24 h on a shaker at 40× *g* and 37 °C. Bacterial culture was diluted by cultivation medium to OD_600_ = 0.1 for the following experiments.

### 3.2. Testing of Antibacterial Properties

Inhibition zones and growth curves were used to test of the antibacterial properties. Petri dishes were covered by 24-h grown culture of non-resistant *S. aureus* and MRSA with 3 mL of LB medium. Circular pieces of fabric (VUP Medical Brno, Brno, Czech Republic) with a diameter of 1 cm were soaked with solutions of ampicillin, oxacillin and penicillin (100 μM) or complexes of selenium nanoparticles (SeNPs) with 100 μM concentration of the same ATBs. The Petri dishes were incubated at 37 °C for 24 h.

The antimicrobial effect of tested compounds was determined by measuring the absorbance using an apparatus Multiskan EX (Thermo Fisher Scientific, Schwerte, Germany). In a microtitration plate, *S. aureus* and MRSA cultures were mixed with ATBs and complexes of SeNPs with ATBs. The total volume in the microtitration plate wells was always 300 µL [21].

### 3.3. Preparation of the SeNPs and Complexes of SeNPs with ATBs

Chitosan at 0.1 g was dissolved in 9 mL of water. Then, 0.1 mL of acetic acid was added with 1 mL of Na_2_SeO_3_·5H_2_O (0.263 g/50 mL) solution. After, the solution was mixed for 1 h. Subsequently, 10 µL of mercaptopropionic acid was added and the solution was stirred for 1 h. Then the pH of the solution was adjusted to 7 by 1 M NaOH (1.4 mL), and the color of the samples became pale orange. The samples were stirred vigorously for 3 h at 25 °C. Then the samples were left at 60 °C for 24 h on magnetic stirrer. After 24 h, the ATBs (ampicillin, oxacillin and penicillin) were added and the final concentrations of ATBs for each complex of SeNPs with ATBs were 1 mM.

### 3.4. Measuring the Biofilm Formed by S. aureus and MRSA Followed by Application of ATBs

The xCELLigence system consists of four main components: the Real time cell analyzer dual plate (RTCA DP), the RTCA DP station, the RTCA computer with integrated software and disposable E-plate 16. Firstly, the optimal seeding concentration for proliferation and RTCA assay of non-resistant *S. aureus* and MRSA were determined. For further measurements, a concentration of 3.7 × 10^7^ CFU/mL was selected. *S. aureus* and MRSA with ATBs (ampicillin, oxacillin and penicillin) were put in to the appropriate wells of E-Plate 16 in concentration of 100 μM in a total volume of 250 μL. The measuring was conducted at 37 °C for 48 h in 15-min intervals.

### 3.5. Gene Expression

#### 3.5.1. Isolation of RNA

Bacterial cultures (1 × 10^8^ of cells) were centrifuged at 6000 rpm and 20 °C for 10 min and the pellets were resuspended in 100 µL of PBS buffer, 100 µL of Tissue Lysis Buffer and 0.1 µL of RNase inhibitors. This volume was pipetted into the sample tube from MagNA Pure Compact RNA Isolation Kit (Roche, Basel, Switzerland), and inserted with other instruments on the appropriate place in the machine. In the second row of the machine, the vials with 20 µL of DNAase were inserted. Next steps were carried out according to the manufacturer’s instructions (“RNA Cell” protocol MagNA).

#### 3.5.2. Reverse Transcription and Amplification of cDNA for *mecA* Gene

The mRNA was converted to cDNA using Transcriptor First Strand cDNA Synthesis Kit (Roche, Basel, Switzerland), using random hexamers. The reaction profile was as follows: 25 °C for 10 min, 55 °C for 30 min and 85 °C for 5 min.

The *mecA* gene was amplified using polymerase chain reaction. The sequences of forward and reverse primers for *mecA* gene were 5′-CCCAATTTGTCTGCCAGTTT-3′, and 5′-TGGCAATATTAACGCACCTC-3′, respectively. The final volume of the PCR reaction mixture was 25 μL containing 17.3 μL of sterile water, 2.5 μL of 1× Taq reaction buffer, 0.5 μL of 100 mM dNTP, 1 μL of forward primer, 1 μL of reverse primer and 0.2 μL of Taq DNA polymerase (Sigma-Aldrich, St. Louis, MO, USA) and 2.5 µL of cDNA. The reaction profile was as follows: 30 cycles of 94 °C for 3 min, 53 °C for 30 s and 72 °C for 30 s and a final extension at 72 °C for 4 min. The amplification was carried out using Mastercycler ep realplex4S (Eppendorf AG, Hamburg, Germany) and a 223 bp fragment for *mecA* gene was obtained.

#### 3.5.3. Visualization and Quantification of Gene Expression

cDNA was mixed with loading buffer and then pipetted into the wells of 1.5% agarose gel with ethidium bromide and the electrophoresis was run in 1× TAE buffer for 90 min at 90 V. The bands were visualized by UV transilluminator at 312 nm (VilberLourmat, Marne-la-Valle´e Cedex, France) and band intensities were quantified and analyzed by Carestream Molecular Imaging Software using *In vivo* Xtreme Imaging System (Rochester, NY, USA) and normalized to *16S* gene.

### 3.6. Determination of Protein Fingerprints by MALDI-TOF

Overnight culture (500 µL, 0.1 OD) was centrifuged at 14,000× *g* for 2 min. The supernatant was discarded and the pellet was suspended in 300 µL of de-ionized water. Then, 900 µL of ethanol was added. After centrifugation at 14,000× *g* for 2 min, the supernatant was discarded and the obtained pellet was air-dried. Then it was dissolved in 25 µL of 70% formic acid (*v*/*v*) and 25 µL of acetonitrile. The samples were centrifuged at 14,000× *g* for 2 min and 1 µL of the clear supernatant was spotted in duplicate onto the MALDI target (MTP 384 target polished steel plate; Bruker Daltonics, Bremen, Germany) and air-dried at a room temperature. Each spot was overlaid with 1 µL of 2,5-dihydroxybenzoic acid matrix solution. The spectra were measured on MALDI-TOF/TOF Bruker in the *m*/*z* range of 2–20 kDa.

### 3.7. The Checkerboard Dilution Test

The antibacterial effects that resulted from combining the two antimicrobial agents were assessed by the checkerboard test. The antimicrobial combination we assayed included selenium nanoparticles and antibiotics (ampicillin, oxacillin, penicillin). The inocula were prepared from colonies that had been grown in LB overnight. The final bacterial concentration after inoculation was 2 × 10^5^ CFU/mL. The MIC was determined after 24 h of incubation at 37 °C. The fractional inhibitory concentration (FIC) index was determined by the formula: FIC index = FIC A + FIC B = [A]/MIC A + [B]/MIC B, where [A] is the concentration of drug A, MICA is its MIC and FICA is the FIC of drug A for the organism, while [B], MICB, and FICB are defined in the same fashion for drug B. The FIC index thus obtained was interpreted as follows: <0.5, synergy; 0.5 to 0.75, partial synergy; 0.76 to 1.0, additive effect; >1.0 to 4.0, indifference; and >4.0, antagonism [47]. Finally, the varying rates of synergy between two agents were determined [48].

### 3.8. Descriptive Statistics

Data were processed using MICROSOFT EXCEL^®^ (Microsoft, Albuquerque, NM, USA) with the pair assay for comparison between sensitive *S. aureus* and methicillin-resistant *S. aureus*. The results are expressed as mean ± standard deviation (S.D.) unless noted otherwise (EXCEL^®^).

## 4. Conclusions

The study was performed to investigate the growth properties and ability to form biofilms in non-resistant *S. aureus* and MRSA after the application of ATBs or complexes of SeNPs with ATBs. Particularly, MRSA, as a common agent of nosocomial infections with resistance to antibiotics, has been the major focus in our investigations of alternatives for inhibition of its growth and multiplication. Our results point to significant antimicrobial effects of SeNPs in with ATBs. The components in the complex can act independently or synergistically and can perform better on a wider spectrum of bacterial species, including antibiotic-resistant species. By the impedance monitoring of biofilms formation revealed that SeNPs can cause biofilm disruption compared with controls without the application of nanoparticles. After the application of nanoparticles or complexes with antibiotics, gene expression was monitored, and was found to be decreasing with increasing concentration of ATBs. The reported results can be used for further experiments concerning ATBs resistance, and especially for the use of selenium nanoparticles as a tool for the treatment of bacterial infection, in the cases where antibiotics are not effective.
